# DHEA and polycystic ovarian syndrome: Meta-analysis of case-control studies

**DOI:** 10.1371/journal.pone.0261552

**Published:** 2021-12-21

**Authors:** Jiby Jolly Benjamin, MaheshKumar K., Teena Koshy, Maruthy K. N., Padmavathi R.

**Affiliations:** 1 Department of Physiology, Sri Ramachandra Medical College and Research Institute, Sri Ramachandra Institute of Higher Education and Research, Porur, Chennai, Tamil Nadu, India; 2 Department of Physiology, Government Yoga and Naturopathy Medical College and Hospital, Chennai, Tamil Nadu, India; 3 Department of Human Genetics, Sri Ramachandra Institute of Higher Education and Research, Porur, Chennai, Tamil Nadu, India; 4 Department of Physiology, Narayana Medical College and Hospital, Nellore, Andra Pradesh, India; China Agricultural University, CHINA

## Abstract

**Background:**

Polycystic ovarian syndrome is a heterogenous endocrine disorder characterized by irregular menstrual cycles, hirsuitism and polycystic ovaries. It is further complicated by metabolic syndrome, infertility and psychological stress. Although the etiopathogenesis is unclear, many studies have pointed out the role of stress in this syndrome. DHEA, being a stress marker is being used by scientists to compare the stress levels between polycystic ovarian cases and healthy controls. However, the results obtained from previous studies are equivocal.

**Objective:**

To perform meta-analysis and find the association between stress and the syndrome.

**Data sources:**

Relevant data till January 2021 were retrieved from PubMed, Scopus, Embase and Web of Science using MeSH terms.

**Study selection:**

Case-control studies having PCOS subjects as cases and healthy women as controls were selected provided; their basal DHEA levels were mentioned in the published articles.

**Data extraction:**

Two authors independently extracted the articles and qualified the final studies.

**Data synthesi:**

Pooled meta-analysis was done using random effect model and showed level of DHEA statistically significant in PCOS compared to healthy controls (SMD = 1.15, 95% CI = 0.59–1.71).Heterogeneity was statistically significant as well (I^2^ = 95%).

**Conclusion:**

Thismeta-analysis on DHEA and PCOS has helped in generating evidence regarding the involvement of stress in the pathogenesis of PCOS.

## Introduction

Polycystic ovarian syndrome (PCOS)is the most common endocrine and metabolic disorder afflicting women belonging to the reproductive age group [1]. This disease was first discovered in United States in the year 1935, when Stein and Leventhal described seven women with typical features of amenorrhoea, hirsuitism and enlarged ovarian cysts [2]. Hence this syndrome is also known as ‘Stein-Leventhal Syndrome’. Since then, incidence of this syndrome is ever increasing to the extent of being a burden in the current community. According to the WHO Statistics taken in 2012, polycystic ovarian syndrome is known to affect 116 million women worldwide [3]. To add on, a statistical report by Futterweit, estimated that 50–75% of PCOS women are unaware that they even have this disease [4]. Apart from having features of anovulation, hyperandrogenism and polycystic ovaries, these women are more prone to develop complications like diabetes mellitus, hypertension, cardiovascular disease, infertility and even carcinomas of ovaries, endometrium and breast [5]. Besides, PCOS women also tend to suffer from psychological symptoms of mood swings, depression and anxiety [6].

Being certain of the increasing prevalence of polycystic ovarian syndrome and its related complications, studying about the cause of these characteristic features is essential. This becomes important as knowing the possible etiology enables physicians to tackle the disease with a better optimal treatment. Many mechanisms have been proposed till date some of which includes dysregulation of steroidogenesis, disordered folliculogenesis, insulin resistance, stress, chronic low-grade inflammation, obesity, genetic linkages, life style choices as well as intrauterine and postnatal environmental factors [7].

One interesting feature women with polycystic ovarian syndrome encompass is psychological morbidity. Studies have found that PCOS women tend to be more anxious and depressed compared to healthy women of their age [8]. They often lead a low quality of life and find it cumbersome to handle stress when they face inconvenience of some kind. Moreover studies have found that PCOS women with emotional distress exhibit clinical symptoms of alopecia, acne, hirsuitism and infertility [9]. Androgen excess is found in almost 70–80% of PCOS women, stating that this syndrome is the leading cause of hirsutism [10].This provides a clear verification that there exists a link between stress, androgen excess and low quality of life.

It is known that acute psychosocial stress increases cortisol release in response to ACTH. As, dehydroepiandrosterone (DHEA) and its sulphated form (DHEA-S) are also released from adrenals in response to ACTH, it is quite logical to regard that the DHEA is also increased in response to acute stress [11]. However, long term psychosocial stress causes a decline in the levels of DHEA and its sulphated form, as it plays a role in resilience and successful adaptation to extreme stress [12].

High levels of DHEA released into the blood stream in response to acute stress have effects on metabolic, endocrine and ovarian functions. Studies have shown that DHEA when induced in rat models stimulates inflammation and oxidative stress causing large follicular cysts to develop in ovaries. This finding mimics human PCOS including added effects of glucose intolerance and androgen excess [13].

Various authors have quantified DHEA, a marker of stress and androgen excess in polycystic ovarian syndrome. As these studies did not depict clear association in any direction, a systematic review and meta-analysis was purposed to study the association of DHEA with PCOS from the existing body of evidence. Similar meta-analyses were also done to find the association of inflammatory genes with female infertility disorders [14] as well as cortisol with PCOS [15].

## Materials and methods

This review was conducted in adherence with the Preferred Reporting Items for Systematic Review and Meta Analyses (PRISMA) statement [16]. The PRISMA checklist is provided in S1 Checklist.

### Literature search and screening

Pubmed, Scopus, Web of Science, Science Direct and Google Scholar were searched till January 2021 using MesH terms. The articles were sought using the following terms and text words in Pubmed “("dehydroepiandrosterone"[MeSH Terms] OR "dehydroepiandrosterone"[All Fields] OR "DHEA"[All Fields]) AND ("poly cystic ovarian syndrome"[MeSH Terms] OR "DHA"[All Fields]) AND ("poly cystic ovarian syndrome"[MeSH Terms] OR " poly cystic ovarian syndrome "[All Fields] OR " Poly cystic ovarian disease"[All Fields] OR " Poly cystic ovarian disease"[All Fields] OR "insulin resistance syndrome"[All Fields] OR “Metabolic Syndrome”[All Fields])”.All articles retrieved were then screened by title or abstract and then by full-text.

### Study selection

Two investigators independently selected and reviewed for relevant case control studies.The selection process was done manually without the aid of any automation tools.

#### Inclusion criteria

Studies were included if (i) the articles were published in English language; (ii) they were of case-control study designs, where cases meant subjects diagnosed with PCOS and controls meant healthy women without PCOS; and (iii) ample data of basal DHEA levels of both cases and controls were provided.

#### Exclusion criteria

Studies were excluded if (i) conducted on animal models; (ii) it was review studies in form of simple review/systematic review/ meta-analysis; (iii) basal DHEA was not measured;(iv) males were included as subjects or controls; (v) samples other than blood were taken for DHEA analysis; (vi) no control group was taken for comparison and (vii) it was purely genetic or prokinetic or in-vitro study.

### Data extraction and quality assessment

Information regarding primary author name, year of publication, country in which the study took place, method of analysis and number of cases and controls were extracted from the studies. Furthermore, all the adequate statistical data on the basal DHEA levels of both the groups were also retrieved. During data extraction, if the first two investigators were not able to arrive at a conclusion, the third investigator resolved any disagreements through discussion.

The quality of the final studies was assessed by Newcastle-Ottawa scale, a scale commonly used for assessing non-randomised studies in meta-analyses (Bouhassira, 2015). The scale ranged from ‘0 to 9’. If a study was scored ≤ 4, it was considered as low-quality study and if it was scored ≥5, it was considered as high-quality study.

### Statistical analysis

Statistical data in the form of Mean ± SD were taken from the cases and control group of the final included studies. For those studies that had data in the form of median, inter-quartile range, standard error and 95% confidence interval (CI), the mean and standard deviation were first calculated from the former, and then taken for meta-analysis, as per a previous paper [17].

Meta-analysis was performed using R software version 3.6.1. By using the random effect model, effect size estimates (standard mean difference [SMD] and 95% confidence intervals [95% CIs]) were calculated [18]. SMD equal to zero was set as the null hypothesis stating no difference in DHEA levels in individuals with PCOS versus controls. Heterogeneity was assessed using a Q test which follows the chi-squared (χ2) distribution and I-squared statistics. If χ2<0.1 or I^2^>50%, the heterogeneity was considered as significant. When heterogeneity was confirmed, possible clarifications were explored post hoc by random-effect subgroup analysis [19]. Outcome of individual studies with overall findingswas graphically displayed by forest plots. A one-way sensitivity analysis was used to verify the results’ robustness.

## Results

A total of 1110 articles were screened and finally 33 studies were included.

### Inclusion process

**Fig 1:**
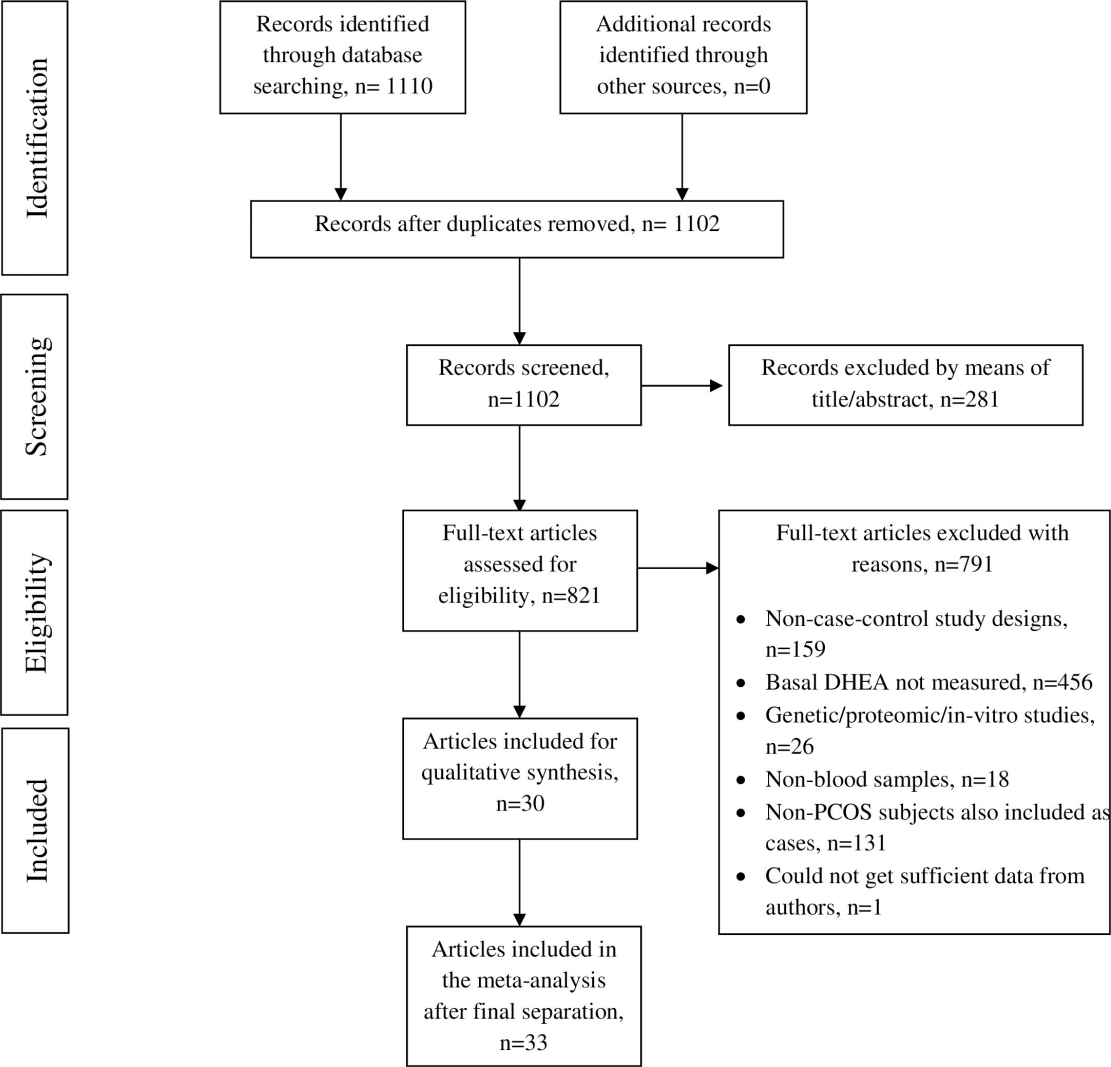
PRISMA flow diagram for the study selection process.

Flow diagram, Fig 1 shows the study selection process of the final articles.

Around 1110 articles were identified through electronic data search. After the duplicates were removed, 1102 studies were screened, of which 281 were removed by means of title or abstract. Further 791 studies were removed from the remaining 821 studies as they did not meet the selection criteria. Finally, 30 studies were qualified for meta-analysis. Three studies were further separated as they had more than one case-control groups. Therefore, the total number of final studies included was 33 articles.

### Systematic review

The essential features of the final studies are depicted in Table 1.

**Table 1:** Essential characteristics of all the included final studies.

Sl.no	Author and year of publication	Country	Sample size (Case/ Control)	DHEA in cases (Mean±SD)	DHEA in controls (Mean±SD)	Unit	Method of analysis	NOSScore
1.	(Abdelazim *et al*., 2020)	Egypt	119/118	8937±2.9	5491±3.7	ng/L	ELISA	6
2.	(Ardawi and Rouzi, 2005)	Saudi Arabia	45/45	27.48±1.87	17.07±1.58	umol/L	EIA	7
3.	(Ardawi and Rouzi, 2005)	Saudi Arabia	45/45	31.72±2.21	19.24±1.5	umol/L	EIA	7
4.	(Buyalos *et al*., 1997)	USA	10/9	4.3± 3.2	3.7± 2.1	ng/mL	RIA	6
5.	(Caanen *et al*., 2016)	UK	14/38	9.11±7.7	6.85±3.76	ng/mL	MS	6
6.	(Cibula *et al*., 2002)	Czech Republic	13/9	12.9±7.3	12.1±3	nmol/L	RIA	6
7.	(de Medeiros *et al*., 2017)	Brazil	147/91	21.8±11.3	18.8±13	nmol/L	ECLIA	6
8.	(Dikensoy *et al*., 2009)	Turkey	60/30	17.1±1.9	14±1.6	nmol/L	PE	5
9.	(Falcone *et al*., 1990)	Canada	19/9	30.2±22.6	28.4±9	nmol/L	RIA	6
10.	(Freitas De Medeiros *et al*., 2020)	Brazil	453/272	15.92±3.29	13.8±3.45	nmol/L	ECLIA	6
11.	(Handelsman *et al*., 2017)	Australia	152/45	7.1±1.72	4.25±2.21	ng/mL	MS	6
12.	(Janse *et al*., 2011)	Netherlands	200/45	15.8±12.17	16±12.54	nmol/L	MS	6
13.	(Liang *et al*., 2020)	China	8/9	7.6±2.4	5.6±2	nmol/L	RIA	6
14.	(Loughlin *et al*., 1986)	Ireland	18/35	14.6±8.4	12.5±6.4	ng/ml	MS	5
15.	(Maas *et al*., 2016)	USA	13/15	1.9±0.2	1.8±1	ng/mL	RIA	6
16.	(Maliqueo *et al*., 2013)	USA	20/30	4±1	2.7±0.45	ng/mL	RIA	7
17.	(Moran *et al*., 1994)	Mexico	6/5	3±0.5	3.7±0.7	ng/mL	RIA	6
18.	(Moran *et al*., 2004)	USA	9/12	23.6±21.22	24.64±12.9	nmol/L	MS	7
19.	(Moran *et al*., 2015)	Mexico	100/16	11.6±7.86	8.4±3.4	ng/mL	RIA	6
20.	(Moran *et al*., 2015)	Mexico	36/26	11.7±10.4	8.4±3.6	ng/mL	RIA	6
21.	(Münzker *et al*., 2015)	UK	275/35	11.2±1.95	5±1.37	ng/mL	MS	6
22.	(O’Reilly *et al*., 2017)	UK	114/49	14.1±1.95	7.1±1.9	nmol/L	MS	5
23.	(Pasquali *et al*., 2007)	Italy	78/21	10.6±7.2	8.1±5.5	μg/mL	RIA	7
24.	(Rahimi and Mohammadi, 2019)	Iran	50/109	278.7±148.7	215.4±142	pg/mL	CL	7
25.	(Rosencrantz *et al*., 2011)	USA	10/11	5.2±4.1	3.5±1.9	ng/mL	RIA	5
26.	(Stener-Victorin *et al*., 2010)	Sweden	74/31	5.2±4.1	3.5±1.9	ng/mL	MS	7
27.	(Tena *et al*., 2011)	Mexico	51/21	6.48±0.81	4.84±0.61	ng/mL	RIA	6
28.	(Turner *et al*., 1992)	UK	50/37	12.2±8.35	10.1±3.57	nmol/L	RIA	8
29.	(Vassiliadi *et al*., 2009)	UK	75/28	59±32	22.7±6.2	nmol/L	RIA	7
30.	(Vassiliadi *et al*., 2009)	UK	103/72	53.2±24	25.1±11.3	nmol/L	RIA	7
31.	(Vrbikova *et al*., 2000)	Czech Republic	24/11	27.6±13.8	19.6±7.3	nmol/L	RIA	7
32.	(Wachs *et al*., 2008)	USA	20/11	2.2±1.05	0.7±0.95	ng/mL	RIA	5
33.	(Yildiz *et al*., 2004)	USA	23/7	5.3±3.5	2.8±1	ng/mL	RIA	6

^b^RIA,Radioimmunoassay; ELISA, Enzyme linked immunosorbent assay; ECLIA,Enzymechemiluminescence linked assay; CL, Chemiluminescence; MS, mass spectrometry; PE, Plasma extraction with ethyl ether.

The 33 case-control studies included a total of 3,781 participants of which 2,434 were PCOS subjects and 1,347 were controls. The incorporated studies were published from the year 1986 to 2020 conducted across 16 countries worldwide. Twelve studies were done in North American continent [20–30], 12 studies in European continent [31–41], 4 studies in Asian continent [42–44], 2 studies in South American continent [45, 46] and 1 study each in Eurasia [47], African [48] and Australian continent [49]. Among these, 18 studies measured DHEA levels by radioimmunoassay [20–24, 26–30, 32, 37, 39–42], 8 studies by mass spectrometry [25, 31, 33–36, 38, 49], 2 studies each by enzyme chemiluminescence linked assay [45, 46] and enzyme linked assay [43] and 1 study each by enzyme linked immunosorbent assay [48], chemiluminescence [44] and plasma extraction with ethyl ether [47]. According to Newcastle Ottawa scale quality score assessment, all 33 studies were rated as ‘high’ quality as all had scores 5 and above; with the highest being ‘8’ and lowest being ‘5’.The Newcastle Ottawa quality score assessment of individual studies is provided in S1 File.

### Pooled meta-analysis

**Fig 2:**
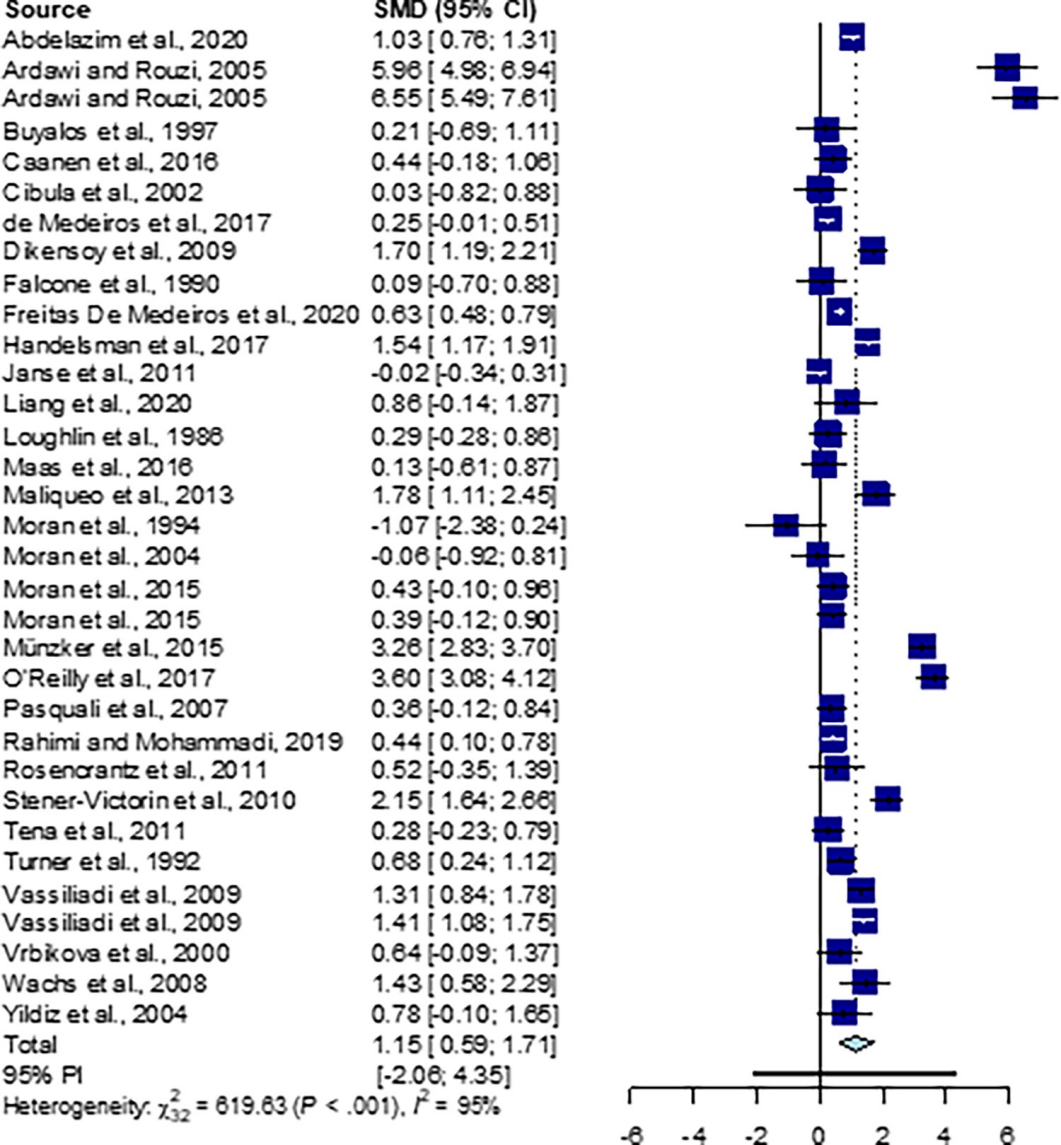
Forest plot of the final 33 studies included in the meta-analysis.

**Fig 3:**
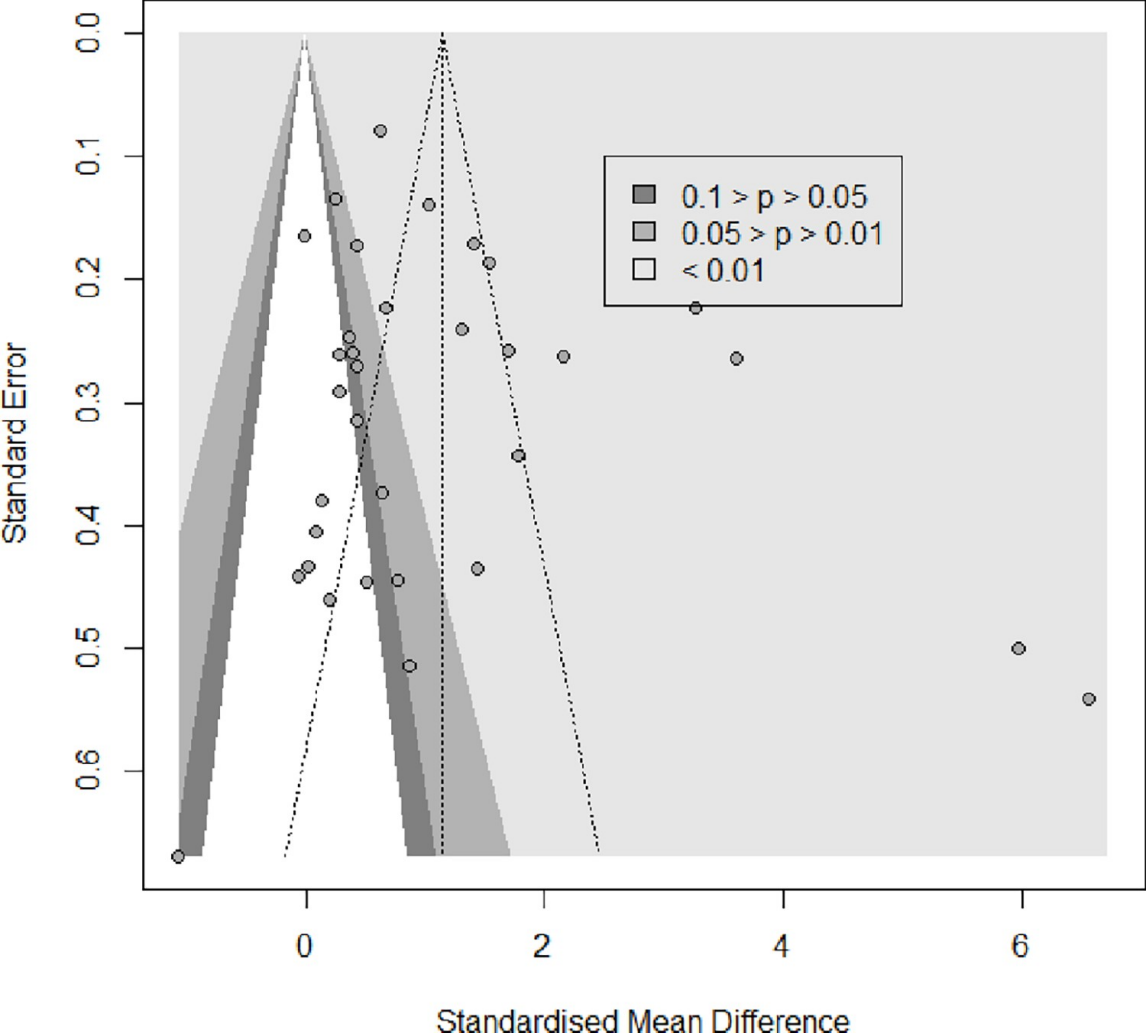
Funnel plot of the final studies qualified in the meta-analysis.

The output of the pooled meta-analysis from 33studies is illustrated in Fig 2. From the pooled data, the level of DHEA was significantly higher in PCOS patients when compared to healthy controls (Random effects, SMD = 1.15, 95% CI = 0.59–1.71, p<0.00001; Fig 2). Moreover heterogeneity across the studies was found to be highly significant (p<0.001, I^2^ = 95%). Asymmetry of the funnel plot and results of Egger’s test showed no evidence of publication bias (p = 0.17) (Fig 3).

### Subgroup and sensitivity analyses

The findings of the subgroup analysis are presented in Table 2. The subgroup analysis suggested that EIA method (SMD-6.23) had significantly more difference on DHEA level compared with other method of assessment. Sensitivity analysis results suggest that there was no change in the summarized results after the exclusion of studies one by one, suggesting that the meta-analysis results were reliable.

**Table 2:** The results of subgroups in the meta-analysis of DHEA levels in PCOS.

	Subgroup	No of studies	SMD	95%-CI	I^2^
**Method of assessment**	**ELISA**	1	1.03	[0.76; 1.30]	--
	**EIA**	2	6.23	[2.50; 9.97]	0.00%
	**RIA**	18	0.62	[0.32; 0.93]	70.70%
	**MS**	8	1.41	[0.18; 2.64]	97.30%
	**ECLIA**	2	0.45	[-1.96; 2.87]	83.50%
	**PE**	1	1.70	[1.19; 2.20]	--
	**CL**	1	0.43	[0.09; 0.77]	--

## Discussion

This meta-analysis was done to find the association of the stress marker DHEA with polycystic ovarian syndrome. From the current literature available on various indexed search engines, the authors could retrieve 1110 articles, of which only 30 studies were included for final analysis. Three studies were further separated as it had more than one case control study. Finally 33 studies were included. These case-control studies measured DHEA levels using various methods of analysis and presented results in different units. This was first of its kind as the authors were unable to find a similar one on association of DHEA in PCOS at the time of data retrieval.

Meta-analysis being a mathematical form of summarisation helps to combine results of similar studies and give specific outcomes [50]. Meta-analysing becomes important as this eliminates unnecessary studies being done which further implies that wasting of resources in terms of time, wealth and man-power can be reduced to a considerable extent. Further, whenstudies done worldwide provide both positive and negative findings, these type of analysis helps to provide a clear insight and conclusion into the same.

In this meta-analysis, pooled estimate stated statistical significant difference where PCOS group was favoured (Fig 1). This suggested an overall elevation of DHEA levels in subjects with PCOS compared to healthy controls. Heterogeneity across the studies also showed statistical significance. This implies that studies that enter a meta-analysis are never identical. The obvious reasons for this is because the studies differ in their sample selection criteria, method adopted to analysis the biomarker, variation in ethnic origin, culture, lifestyle choices and food habits among participants from various parts of the world. In terms of magnitude, the heterogeneity obtained is high scale, as I^2^ is more than 75%. As heterogeneity was statistically significant, we proceeded in doing Egger’s test to verify whether it was due to publication bias. The results of the regression test showed no evidence of publication bias as the intercept departs from zero (Fig 3).

Under sub group analysis based on type of method assessment, EIA had the highest standardized mean difference compared to other methods. It thus assured that EIA had a bigger intervention effect in relation to variability observed in study. In order to rely on the results of the meta-analysis, we also conducted sensitivity analysis. It showed no change in the final result even after removing each study serially.

DHEA was chosen as a biomarker for this meta-analysis, with a reason to assess the significance of stress in this syndrome. DHEA is an important stress marker released commonly from the adrenal cortex in response to ACTH when provoked by a stressor [51]. Physiologically, circulating DHEA is also produced and released in small amounts from the ovaries and testis [11]. However, in PCOS women with hirsutism, the major source of androgen production is from the ovaries rather than adrenals [52]. This can be explained by theca cell defect in polycystic ovaries causing over expression of steroidogenic enzymes particularly CYP17A1 enzyme that enhances DHEA production [7]. Increased DHEA reflects psychosocial stress and exhibits clinically in the form of anxiety and depression. Also excess androgens interfere with female hormone synthesis and cause symptoms from milder ones like acne to severe symptoms like infertility [7]. PCOS women with infertility tend to be more anxious and this often sparks into a vicious cycle where increased anxiety and stress worsens infertility and vice-versa. Moreover, these women tend to adopt unhealthy eating behaviours and sedentary lifestyle so as to cope up with the psychological distress and emotional trauma [53]. And they often find themselves suffering from adverse complications of obesity as well. The result of this meta-analysis well supports and goes hand-in-hand with the pathomechanism of elevated DHEA seen in PCOS.

Other stress markers have also been studied under meta-analysis in PCOS. A meta-analysis that linked cortisol with PCOS showed overall elevation of cortisol levels in PCOS subjects [15]. This suggested that stress was appreciably related to this disease. However, as controversial findings have been reported on association of stress markers DHEA and cortisol in individual PCOS studies, a more reliable marker would be to consider DHEA-cortisol ratio [54]. This marker could potentially strengthen the impact of stress in this disease.

Another angle to confirm whether stress is involved in the pathophysiological mechanism of PCOS is to observe for alleviation of symptoms on administration of anti-oxidants. Oxidative stress at cellular level can be reduced or prevented with the aid of anti-oxidants. Vitamin D, a powerful antioxidant is being used by scientists to check for the ease of symptoms in those suffering from PCOS. A meta-analysis that studied the effect of Vitamin D on oxidative stress in PCOS subjects suggested improved oxidative stress levels following treatment with Vitamin D [55].

One major advantage of doing this meta-analysis on DHEA and PCOS is that it helped to provide clear insight on association of DHEA in this syndrome based on marker analysis. It also helped to overcome the limitations of individual studies with small sample size. Moreover, the statistical power of this analysis was considerably noticeable. But at the same time, few limitations did exist in this study. There were variations in methodological characteristics in the studies while measuring serum DHEA levels which could result in detection bias. There were studies that were published in different languages and some had insufficient data which if included may have had the potential to strengthen the existing results.

## Conclusion

This meta-analysis was able to provide apparent close by into the association of DHEA with PCOS by suggesting a significant elevation of DHEA levels in women with this syndrome compared to healthy controls. Overall, the analysis supports the findings of increased DHEA in PCOS. In future, more research is recommended from Asian countries with regard to this field of medicine.

## Supporting information

S1 Checklist PRISMA checklist. (DOCX)

S1 File Newcastle Ottawa scale scoring case-control studies. (DOCX)

S1 Table Details of data included in the meta analysis. (DOCX)
