# Supplementary material for: DHEA and polycystic ovarian syndrome: Meta-analysis of case-control studies
Source: PLoS One. 2021 Dec 21;16(12):e0261552. doi: 10.1371/journal.pone.0261552 (PMC8691613; doi:10.1371/journal.pone.0261552)
Supplement: S1 File — (DOCX) [file pone.0261552.s002.docx]

| **Sl.no** | **Author and year of publication** | **SELECTION**  **(1)** | **SELECTION**  **(2)** | **SELECTION**  **(3)** | **SELECTION**  **(4)** | **COMPARRABILBITY**  **(1)** | **EXPOSURE**  **(1)** | **EXPOSURE**  **(2)** | **EXPOSURE**  **(3)** | **TOTAL SCORE** |
| --- | --- | --- | --- | --- | --- | --- | --- | --- | --- | --- |
| 1. | (Abdelazim *et al.*, 2020) | * | -- | -- | * | * -- | * | * | * | **6** |
| 2. | (Ardawi and Rouzi, 2005) | * | * | -- | * | * -- | * | * | * | **7** |
| 3. | (Ardawi and Rouzi, 2005) | * | * | -- | * | * -- | * | * | * | **7** |
| 4. | (Buyalos *et al.*, 1997) | * | -- | -- | * | * -- | * | * | * | **6** |
| 5. | (Caanen *et al.*, 2016) | * | -- | -- | * | * -- | * | * | * | **6** |
| 6. | (Cibula *et al.*, 2002) | * | -- | -- | * | * -- | * | * | * | **6** |
| 7. | (de Medeiros *et al.*, 2017) | * | -- | -- | * | * -- | * | * | * | **6** |
| 8. | (Dikensoy *et al.*, 2009) | * | -- | -- | * | * -- | * | * | -- | **5** |
| 9. | (Falcone *et al.*, 1990) | * | -- | -- | * | * -- | * | * | * | **6** |
| 10. | (Freitas De Medeiros *et al.*, 2020) | * | -- | -- | * | * -- | * | * | * | **6** |
| 11. | (Handelsman *et al.*, 2017) | * | -- | -- | * | * -- | * | * | * | **6** |
| 12. | (Janse *et al.*, 2011) | * | -- | -- | * | * -- | * | * | * | **6** |
| 13. | (Liang *et al.*, 2020) | * | -- | -- | * | * -- | * | * | * | **6** |
| 14. | (Loughlin *et al.*, 1986) | * | -- | -- | -- | * -- | * | * | * | **5** |
| 15. | (Maas *et al.*, 2016) | * | -- | -- | * | * -- | * | * | * | **6** |
| 16. | (Maliqueo *et al.*, 2013) | * | * | -- | * | * -- | * | * | * | **7** |
| 17. | (Moran *et al.*, 1994) | * | -- | -- | * | * -- | * | * | * | **6** |
| 18. | (Moran *et al.*, 2004) | * | -- | * | * | * -- | * | * | * | **7** |
| 19. | (Moran *et al.*, 2015) | * | -- | -- | * | * -- | * | * | * | **6** |
| 20. | (Moran *et al.*, 2015) | * | -- | -- | * | * -- | * | * | * | **6** |
| 21. | (Münzker *et al.*, 2015) | * | -- | -- | * | * -- | * | * | * | **6** |
| 22. | (O’Reilly *et al.*, 2017) | * | -- | -- | -- | * -- | * | * | * | **5** |
| 23. | (Pasquali *et al.*, 2007) | * | * | -- | * | * -- | * | * | * | **7** |
| 24. | (Rahimi and Mohammadi, 2019) | * | -- | * | * | * -- | * | * | * | **7** |
| 25. | (Rosencrantz *et al.*, 2011) | * | -- | -- | -- | * -- | * | * | * | **5** |
| 26. | (Stener-Victorin *et al.*, 2010) | * | -- | * | * | * -- | * | * | * | **7** |
| 27. | (Tena *et al.*, 2011) | * | -- | -- | * | * -- | * | * | * | **6** |
| 28. | (Turner *et al.*, 1992) | * | * | * | * | * -- | * | * | * | **8** |
| 29. | (Vassiliadi *et al.*, 2009) | * | -- | * | * | * -- | * | * | * | **7** |
| 30. | (Vassiliadi *et al.*, 2009) | * | -- | * | * | * -- | * | * | * | **7** |
| 31. | (Vrbikova *et al.*, 2000) | * | -- | * | * | * -- | * | * | * | **7** |
| 32. | (Wachs *et al.*, 2008) | * | -- | -- | -- | * -- | * | * | * | **5** |
| 33. | (Yildiz *et al.*, 2004) | * | -- | -- | * | * -- | * | * | * | **6** |

CODING MANUAL FOR CASE-CONTROL STUDIES

***SELECTION***

1. **Is the Case Definition Adequate?**
2. Requires some independent validation (e.g. >1 person/record/time/process to extract information, or reference to primary record source such as x-rays or medical/hospital records)
3. Record linkage (e.g. ICD codes in database) or self-report with no reference to primary record
4. No description
5. **Representativeness of the Cases**
6. All eligible cases with outcome of interest over a defined period of time, all cases in a defined catchment area, all cases in a defined hospital or clinic, group of hospitals, health maintenance organisation, or an appropriate sample of those cases (e.g. random sample)
7. Not satisfying requirements in part (a), or not stated.
8. **Selection of Controls**

This item assesses whether the control series used in the study is derived from the same population as the cases and essentially would have been cases had the outcome been present.

1. Community controls (i.e. same community as cases and would be cases if had outcome)
2. Hospital controls, within same community as cases (i.e. not another city) but derived from a hospitalised population
3. No description
4. **Definition of Controls**
5. If cases are first occurrence of outcome, then it must explicitly state that controls have no history of this outcome. If cases have new (not necessarily first) occurrence of outcome, then controls with previous occurrences of outcome of interest should not be excluded.
6. No mention of history of outcome

# COMPARABILITY

1. **Comparability of Cases and Controls on the Basis of the Design or Analysis**

## A maximum of 2 stars can be allotted in this category

Either cases and controls must be matched in the design and/or confounders must be adjusted for in the analysis. Statements of no differences between groups or that differences were not statistically significant are not sufficient for establishing comparability. Note: If the odds ratio for the exposure of interest is adjusted for the confounders listed, then the groups will be considered to be comparable on each variable used in the adjustment.

There may be multiple ratings for this item for different categories of exposure (e.g. ever vs. never, current vs. previous or never)

Age = , Other controlled factors =

# EXPOSURE

1. **Ascertainment of Exposure**

Allocation of stars as per rating sheet

1. **Non-Response Rate**

Allocation of stars as per rating sheet
