# Supplementary material for: DHEA and polycystic ovarian syndrome: Meta-analysis of case-control studies
Source: PLoS One. 2021 Dec 21;16(12):e0261552. doi: 10.1371/journal.pone.0261552 (PMC8691613; doi:10.1371/journal.pone.0261552)
Supplement: S1 Table — (DOCX) [file pone.0261552.s003.docx]

| **AUTHOR AND YEAR OF PUBLICATION** | **COUNTRY** | **Experimental group (Mean)** | **Experimental group (SD/range)** | **Experimental group (No of participants )** | **Control group (Mean)** | **Control group (SD/range)** | **Control group (No of participants )** | **METHOD OF ANALYSIS** | **UNIT** |
| --- | --- | --- | --- | --- | --- | --- | --- | --- | --- |
| Abdelazim *et al.*, 2020 | Egypt | 8937 | 2.9 | 119 | 5491 | 3.7 | 118 | ELISA | ng/L |
| Ardawi and Rouzi, 2005 | Saudi Arabia | 27.48 | 1.87 | 45 | 17.07 | 1.58 | 45 | EIA | umol/L |
| Ardawi and Rouzi, 2005 | Saudi Arabia | 31.72 | 2.21 | 45 | 19.24 | 1.5 | 45 | EIA | umol/L |
| Buyalos *et al.*, 1997 | USA | 4.3 | 3.2 | 10 | 3.7 | 2.1 | 9 | Radioimmunoassay | ng/ml |
| Caanen *et al.*, 2016 | United Kingdom | 9.11 | 7.7 | 14 | 6.85 | 3.76 | 38 | Mass spectrometry | ng/ml |
| Cibula *et al.*, 2002 | Czech Republic | 12.3 | 2.2 | 13 | 12.1 | 1 | 9 | Radioimmunoassay | nmol/L |
| de Medeiros *et al.*, 2017 | Brazil | 21.8 | 11.3 | 147 | 18.8 | 13 | 91 | ECLIA | nmol/L |
| Dikensoy *et al.*, 2009 | Turkey | 17.1 | 1.9 | 60 | 14 | 1.6 | 30 | Plasma extraction with ethyl ether | nmol/L |
| Falcone *et al.*, 1990 | Canada | 30.2 | 5.2 | 19 | 28.4 | 3 | 9 | Radioimmunoassay | nmol/L |
| Freitas De Medeiros *et al.*, 2020 | Brazil | 15.92 | 9.85–23.04 | 453 | 13.8 | 8.51–22.38 | 272 | ECLIA | nmol/L |
| Handelsman *et al.*, 2017 | Australia | 7.1 | 0.4 | 152 | 4.25 | 0.33 | 45 | Mass spectrometry | ng/mL |
| Janse *et al.*, 2011 | Netherlands | 15.8 | 4.34–65.2 | 200 | 16 | 8.74–58.9 | 45 | Mass spectrometry | nmol/L |
| Liang *et al.*, 2020 | China | 7.6 | 2.4 | 8 | 5.6 | 2 | 9 | Radioimmunoassay | mmol/L |
| Loughlin *et al.*, 1986 | Ireland | 14.6 | 8.4 | 18 | 12.5 | 6.4 | 35 | Mass spectrometry | ng/ml |
| Maas *et al.*, 2016 | USA | 1.9 | 0.2 | 13 | 1.8 | 1 | 15 | Radioimmunoassay | ng/mL |
| Maliqueo *et al.*, 2013 | USA | 4 | 1.9–5.9 | 20 | 2.7 | 1.9–4.4 | 30 | Radioimmunoassay | ng/mL |
| Moran *et al.*, 1994 | Mexico | 3 | 0.5 | 6 | 3.7 | 0.7 | 5 | Radioimmunoassay | ng/mL |
| Moran *et al.*, 2004 | USA | 23.6 | 12.15–97.16 | 9 | 24.64 | 5.21–57.26 | 12 | Mass spectrometry | nmol/L |
| Moran *et al.*, 2015 | Mexico | 11.6 | 2.2–33.6 | 100 | 8.4 | 4.3–17.9 | 16 | Radioimmunoassay | ng/mL |
| Moran *et al.*, 2015 | Mexico | 11.7 | 4.1–45.7 | 36 | 8.4 | 2.4–16.8 | 26 | Radioimmunoassay | ng/mL |
| Münzker *et al.*, 2015 | United Kingdom | 11.2 | 7.7-15.5 | 275 | 5 | 4-9.5 | 35 | Mass spectrometry | ng/mL |
| O’Reilly *et al.*, 2017 | United Kingdom | 14.1 | 10.4–18.2 | 114 | 7.1 | 4.2–11.8 | 49 | Mass spectrometry | nmol/L |
| Pasquali *et al.*, 2007 | Italy | 10.6 | 7.2 | 78 | 8.1 | 5.5 | 21 | Radioimmunoassay | µg/mL |
| Rahimi and Mohammadi, 2019 | Iran | 278.7 | 148.7 | 50 | 215.4 | 142 | 109 | Chemiluminescence | pg/mL |
| Rosencrantz *et al.*, 2011 | USA | 5.2 | 4.1 | 10 | 3.5 | 1.9 | 11 | Radioimmunoassay | ng/mL |
| Stener-Victorin *et al.*, 2010 | Sweden | 6.48 | 5.08–8.34 | 74 | 4.84 | 3.82–6.28 | 31 | Mass spectrometry | ng/mL |
| Tena *et al.*, 2011 | Mexico | 12.2 | 2.2–35.6 | 51 | 10.1 | 5.8–20.1 | 21 | Radioimmunoassay | ng/mL |
| Turner *et al.*, 1992 | United Kingdom | 33.5 | 14-90 | 50 | 23 | 9.7-40.1 | 37 | Radioimmunoassay | nmol/L |
| Vassiliadi *et al.*, 2009 | United Kingdom | 59 | 32 | 75 | 22.7 | 6.2 | 28 | Radioimmunoassay | nmol/L |
| Vassiliadi *et al.*, 2009 | United Kingdom | 53.2 | 24 | 103 | 25.1 | 11.3 | 72 | Radioimmunoassay | nmol/L |
| Vrbikova *et al.*, 2000 | Czech Republic | 27.6 | 13.8 | 24 | 19.6 | 7.3 | 11 | Radioimmunoassay | nmol/L |
| Wachs *et al.*, 2008 | USA | 2.2 | 1.05 | 20 | 0.7 | 0.95 | 10 | Radioimmunoassay | ng/mL |
| Yildiz *et al.*, 2004 | USA | 5.3 | 3.5 | 23 | 2.8 | 1 | 7 | Radioimmunoassay | ng/mL |
|  |  |  | IQR | Median | SE |  |  |  |  |
